# National Association of Dental Faculties

**Published:** 1890-09

**Authors:** 


### NATIONAL ASSOCIATION OF DENTAL FACULTIES.

This Association convened at Excelsior Springs, Mo., August 4.

The report of subjects claiming its attention, and of general interest, is given in another part of this journal.

The meeting was an exceedingly harmonious one, notwithstanding previous attempts to stir up disturbing elements. The feeling seemed to prevail that the work to be done was to complete that of the previous year (1889), and in no way to unsettle the decisions of that meeting.

The problem of graded courses was met and thoroughly discussed; but as it was manifestly impossible to meet all the peculiar conditions surrounding the various schools, it was wisely concluded to simply recommend certain changes, leaving their adoption to the judgment of the separate faculties. A similar course was adopted in regard to the equalization of fees. The reasons for this action do not seem as clear as in the former case. It would seem that the time had arrived to make this mandatory, all the more as there appeared no good reason why this course should not have been adopted.

The schools belonging to the Association were generally represented by delegates,—a few by letter. The proceedings throughout manifested a healthful spirit of honest determination to advance the standard of dental education steadily and persistently, but, at the same time, with a due regard to the best interest of the students and colleges.

